# Direct and indirect association of domestic violence against women and severe maternal morbidity: A case–control study

**DOI:** 10.1097/MD.0000000000041268

**Published:** 2025-01-24

**Authors:** Mahdieh Sahebi, Sedigheh Abdollahpour, Masoumeh Sadeghi, Hamid Heidarian Miri

**Affiliations:** a Department of Epidemiology, School of Health, Mashhad University of Medical Sciences, Mashhad, Iran; b Nursing and Midwifery Care Research Center, Mashhad University of Medical Sciences, Mashhad, Iran; c School of Nursing and Midwifery, University College Cork, Cork, Ireland.

**Keywords:** domestic violence, mediation analysis, severe maternal morbidity

## Abstract

This study aimed to investigate the direct association between domestic violence and the indirect association of exposure through pregnancy, delivery, and neonatal risk factors with severe maternal morbidity (SMM). The target population of this case–control study included all women who gave birth in the hospitals of the Torbat Heidarieh University of Medical Science from June 2018 to May 2020. A total of 123 mothers with SMM according to the World Health Organization criteria were selected as cases, and 127 mothers who did not meet the World Health Organization criteria were included in the control group. Data were analyzed using Stata 14 and mediation packages with a counterfactual approach. The odds ratio of the pure direct effect of physical violence on SMM through pregnancy and neonatal risk factors is 2.26 and 2.29, respectively. The odds ratio of the pure direct effect of social violence on SMM through pregnancy, delivery, and neonatal risk factors was 2.54, 2.67, and 2.57, respectively, and that for economic violence through neonatal risk factors was 1.99. Additionally, the interaction between physical and social violence and pregnancy risk factors increased the risk of severe maternal morbidity by 394.6% and 360%, respectively. Domestic violence against women is directly associated with severe maternal morbidity. Physical and social violence showed a significant interaction with severe maternal morbidity. A preventive program for domestic violence should be considered an effective intervention to prevent severe maternal morbidity and improve the health of mothers by implementing control strategies.

## 1. Introduction

Along with the reduction in maternal mortality, there has been increasing attention on severe maternal morbidity (SMM) for evaluating maternal health programs. According to the World Health Organization (WHO), these morbidities have 2 stages: potential life-threatening conditions and life-threatening conditions. In the second stage, the mother experiences organ dysfunction. If a mother survives life-threatening conditions but experiences organ dysfunction during pregnancy, delivery, and the 6-week postpartum period, she is classified as a maternal near miss (MNM). Otherwise, maternal death may have occurred. Postpartum hemorrhage, severe preeclampsia, eclampsia, sepsis, and uterine rupture are factors related to the first stage. Factors related to MNMs include cardiovascular, respiratory, renal, coagulation/hematological, hepatic, neurological, and uterine dysfunction.^[1,2]^

To understand severe maternal morbidity, it is important to be aware of the risk factors that are similar to those associated with maternal mortality. The WHO has identified hemorrhage, hypertensive disorders, sepsis, and obstructed labor as the 4 main risk factors for maternal mortality.^[3]^ Hemorrhage and hypertensive disorders are important risk factors in low-income, middle-income, and high-income countries.^[4]^ Additionally, other risk factors are considered, such as social determinants of sexual violence and domestic violence, which are associated with maternal mortality and severe maternal morbidity, respectively.^[5,6]^

The global prevalence of severe maternal morbidity according to WHO criteria is 18.67/1000 live births. By continent, the prevalence of this indicator is 3.10, 31.88, 11.57, 17.14, and 16 in Europe, Africa, South America, Oceania, and 16.92 in Asia per 1000 live births.^[7]^ The ratio of severe in Iran is 2.50/1000 in Iran.^[8]^ According to a WHO report, 1 in 3 women has experienced physical or sexual intimate partner violence or non-partner sexual violence in their lifetime. Additionally, 27% of women between 15 and 49 years of age who have been in a relationship experienced physical and sexual violence from their intimate partner.^[9]^ In an Iranian meta-analysis that included 15,514 participants, the prevalence of women experiencing domestic violence was estimated to be 66%. The prevalence of domestic violence in the east and south of Iran was 70%, 75% in the west, and 62% in the north of Iran.^[10]^

Social determinants such as violence can be directly or indirectly associated with SMM through pregnancy, delivery, or neonatal risk factors. It is important to care about the psychological condition of MNM/SMM mothers besides the physical life by maternal health providers.^[11]^ Most studies have utilized ordinary regression models to detect the associations between exposure and outcomes.^[6,11,12]^ However, it is recommended that we employ an analysis that can identify the extent of this association and account for the role of mediators. Given the importance of this indicator in assessing the quality of maternal healthcare and the WHO protocol for accurately detecting severe maternal morbidity cases, our study aimed to comprehend the different roles of variables and their direct and indirect associations with severe maternal morbidity.

## 2. Method

### 2.1. Data

We conducted a case–control study using data obtained from women who gave birth at the hospitals of the Torbat Heidarieh University of Medical Sciences from June 2018 to May 2020.

Sample size calculations indicated that to detect an odds ratio of 2 with 90% power and a 95% confidence level in a case–control design, assuming a 0.41 probability of exposure, 122 cases and 122 controls would be required. To account for potential nonparticipation, this number was increased to 130 per group. These input parameters were derived from a pilot study. However, post hoc power analyses based on actual findings revealed that the study’s power to detect the marginal total effect of total violence in the mediation analysis was approximately 50%. Sample size calculations and power analyses were conducted using PASS.

In this study, the cases consisted of women who have experienced severe maternal morbidity or life-threatening conditions during pregnancy, childbirth, and 42 days postpartum according to the WHO including those suffering from the following: severe preeclampsia, eclampsia, severe postpartum hemorrhage, blood transfusion of more than 5 units, sepsis or severe systemic infection ruptured uterus, severe complications of abortion, septic shock, admission to intensive care unit, cardiovascular dysfunction, respiratory dysfunction, renal dysfunction, coagulation/hematological dysfunction, hepatic dysfunction, neurological dysfunction, uterine dysfunction: hysterectomy due to uterine hemorrhage or infection.^[1]^

On the other hand, the controls were mothers who did not experience any complications throughout their pregnancy or childbirth and had a normal delivery.

Written informed consent was obtained from all participants. Ethical approval was obtained from the Mashhad University of Medical Sciences. It was IR.MUMS.FHMPM.REC.1400.107.

### 2.2. Exposure and mediators

In this study, violence and its subgroups were considered as exposure, and pregnancy, delivery, and neonatal risk factors were mediators.

The researchers visited the hospitals every morning to collect demographic data and information on outcomes and mediators from patient files. They also used the domestic violence against women questionnaire, which was obtained from The Mother and Infant System of the Ministry of Interior of Iran. This questionnaire consists of 30 questions, including 9 on mental violence, 9 on physical violence, 5 on social violence, 4 on economic violence, and 3 on sexual violence. Each question had 5 response options, never, rarely, occasionally, frequently, and very frequently, which were assigned scores ranging from zero to 4, respectively. Because each subgroup had a different number of questions, the score for each subgroup was divided by the maximum possible score within that subgroup. For example, scores for mental and physical violence were divided into 36 categories: social violence 20, economic violence 16, and sexual violence, 12. The mean score is calculated based on these scores.^[12]^ The reliability coefficient of the questionnaire is 85%.^[13]^

In this study, pregnancy, delivery, and neonatal risk factors are chosen based on the Iranian Ministry of Health.^[14]^ Pregnancy risk factors included chronic hypertension, cardiovascular disease, non-gestational diabetes, hypothyroidism, hepatitis, HIV, chorioamnionitis, urinary infection, gestational diabetes, preeclampsia, eclampsia, hemoglobin <7 at the time of hospitalization, smoking, tobacco use, drug addiction, and alcohol consumption. Mothers who met at least 1 of these criteria were identified as having pregnancy risk factors.

Delivery risk factors include premature rupture of the amnion for more than 18 hours, placental abruption, meconium contamination, placenta accreta, abnormal heart rate, premature labor, receiving blood products, 3rd and 4th-degree rupture, dystocia, episiotomy, induction of labor, use of vacuum, use of spinal or epidural analgesia, and finally maternal (postpartum ward, operating room, intensive care). Every mother who has at least one of these criteria is identified as having delivery risk factors.

Neonatal risk factors included sex, weight, height, head circumference, gestational age at birth, type of delivery, presentation, number of babies, Apgar score, progression of resuscitation stages, delivery method, postnatal care (mother–infant care, hospitalization, stillbirth, newborn care, and transfer to another hospital), and infant delivery injuries. If a baby has any of these characteristics in an abnormal state, it is considered to have neonatal risk factors.

### 2.3. Confounders

These variables were checked for confounding in the mediation analysis model: mother’s age, pregnancy risk factors (for delivery and neonatal risk factors), delivery risk factors (for pregnancy and neonatal risk factors), neonatal risk factors (for pregnancy and delivery risk factors), number of pregnancies, number of live births, mother’s job, abortion, stillbirth, address (living in a city or village), and family relationship with the spouse.

Finally, the adjusted model included these variables as confounders with a *P*-value < .2: pregnancy risk factors, delivery risk factors, neonatal risk factors, number of pregnancies, number of live births, mother’s age, address, and family relationship with the spouse.

### 2.4. Statistical analysis

In this study, we used Baron and Kenny criteria for mediation analysis with a counterfactual approach, which employs a parametric approach to direct and indirect effects. These criteria introduced the following regression model (Equation 1):


$$\begin{array}{l} \left(M|A=a,C=c\right)=\beta_{0+}\beta_{1}a_{+}{\beta_{2}}^{}c \end{array}$$

$$\begin{array}{l} \left(Y|A=a,M=m,C=c\right)=\theta_{0+}\theta_{1}a_{+}\theta_{2}m_{+}{\theta_{4}}^{} \end{array}$$

Equation 1: regression model for mediator and outcome in mediation analysis consists of 2 parts: 1 for the mediator and the other for the outcome. In this model, we assume that A represents the exposure, Y represents the outcome, M represents the mediator, and C represents the covariates. The direct effect is estimated using θ1, which is the exposure coefficient in the outcome model (second model). The indirect effect is estimated using 2θ1β, which is the product of the exposure coefficient in the mediator model and the mediator coefficient in the outcome model.^[15]^ If we use the counterfactual approach, we can estimate both the natural and controlled effects. Natural effects can be further divided into pure and total effects, based on the presence of interactions.

The analysis was conducted using Paramed and Med4way in Stata 14.^[16,17]^ Variables such as violence, pregnancy, delivery, and neonatal risk factors were considered to be binary. We adjusted for confounders in both packages. The results were reported with a 95% confidence interval and a *P*-value <.05 for odds ratio and excess relative risk. Further details regarding the definition of mediation effects are reported in the Supplement 1 (see Supplement 1, Supplemental Digital Content, http://links.lww.com/MD/O286, which define causal mediation effects).

## 3 Results

### 3.1. Descriptive findings

The mean age of the control group was 28.60 years, while that of the case group was 27.30 years. In the case group, 36.59% were aged 15 to 24.9 years, compared to 40.16% in the control group. Furthermore, 69.92% of the cases and 54.33% of the controls resided in the village (Table 1).

**Table 1:** Demographic characteristics of severe maternal morbidity cases and controls.

Variables		Case (n: 123)		Controls (n: 127)	
		Frequency	Percent (%)	Frequency	Percent (%)
Age group (year)	15–24.9	45	36.59	51	40.16
	25–29.9	27	21.95	29	22.83
	30–34.9	22	17.89	24	18.90
	35–46	29	23.58	12	18.11
Residential area	Urban	37	30.08	58	45.67
	Village	86	69.92	69	54.33
Employment status	Employed	3	2.44	6	4.72
	Housewife	120	97.56	121	95.28
Children	<2 children	71	57.75	83	65.35
	More than 2 children	52	42.28	44	34.65
Family relationship with the spouse	Yes	13	10.57	7	5.51
	No	110	89.43	120	94.49
History of abortion	<2 times	122	99.19	126	99.21
	More than 2 times	1	0.81	1	0.79
History of stillbirth	Yes	4	3.25	4	3.15
	No	119	96.75	123	96.85

More information about the distribution of domestic violence and its subgroups and mediators is provided in the Supplements 2 and 3 (see Tables S2 and S3, Supplemental Digital Content, http://links.lww.com/MD/O286, which illustrate the distribution of cases and controls based on the exposure and its subgroup and mediators).

### 3.2. Analytical findings

According to Table 2, the odds ratio of the pure direct effect and marginal total effect of physical violence on severe maternal morbidity with is 2.26 (*P* = .048) and 2.30 (*P* = .029) through pregnancy risk factors (mediator), respectively. The odds ratio of the controlled and pure direct effect and the marginal total effect of physical violence on severe maternal morbidity through neonatal risk factors are 2.42 (*P* = .043), 2.29 (*P* = .025), and 2.15 (*P* = .035) respectively. These significant effects demonstrate a direct effect of physical violence on severe maternal morbidity.

**Table 2:** The results of mediation analysis with Paramed package for violence and its subgroup and severe maternal morbidity.

		CDE			PDE			TIE			MTE		
		OR	95% CI	*P*-value	OR	95% CI	*P*-value	OR	95% CI	*P*-value	OR	95% CI	*P*-value
Total violence	Pregnancy risk factors	0.47	(0.15, 1.46)	.193	1.11	(0.54, 2.27)	.766	1.26	(0.92, 1.71)	.148	1.40	(0.63, 3.10)	.406
	Delivery risk factors	1.06	(0.31, 3.59)	.921	1.20	(0.64, 2.22)	.562	0.91	(0.74, 1.12)	.403	1.10	(0.59, 2.02)	.763
	Neonatal risk factors	1.58	(0.78, 3.26)	.203	1.36	(0.74, 2.48)	.323	0.96	(0.83, 1.09)	.541	1.30	(0.71, 2.37)	.395
Mental violence	Pregnancy risk factors	0.55	(0.18, 1.67)	.293	0.63	(0.33, 1.17)	.147	1.27	(0.86, 1.87)	.215	0.80	(0.37, 1.70)	.564
	Delivery risk factors	0.55	(0.16, 1.82)	.330	0.64	(0.35, 1.17)	.148	1.06	(0.89, 1.25)	.495	0.68	(0.36, 1.27)	.229
	Neonatal risk factors	0.79	(0.38, 1.60)	.514	0.68	(0.36, 1.26)	.221	0.94	(0.80, 1.10)	.451	0.64	(0.34, 1.19)	.158
Physical violence	Pregnancy risk factors	0.47	(0.15, 1.45)	.189	**2.26**	**(1.00, 5.08)**	**.048**	1.01	(0.72, 1.42)	.933	**2.30**	**(1.08, 4.84)**	**.029**
	Delivery risk factors	1.17	(0.24, 5.58)	.839	1.94	(0.97, 3.85)	0.058	0.97	(0.84, 1.11)	.711	1.89	(0.95, 3.75)	.068
	Neonatal risk factors	**2.42**	**(1.02, 5.71)**	**.043**	**2.29**	**(1.10, 4.73)**	**.025**	0.94	(0.78, 1.11)	.486	**2.15**	**(1.05, 4.39)**	**.035**
Social violence	Pregnancy risk factors	0.60	(0.18, 1.93)	.396	**2.54**	**(1.13, 5.70)**	**.024**	1.04	(0.72, 1.50)	.824	**2.65**	**(1.23, 5.70)**	**.013**
	Delivery risk factors	2.96	(0.51, 16.97)	.224	**2.67**	**(1.14, 6.22)**	**.023**	0.84	(0.53, 1.30)	.431	**2.24**	**(1.06, 4.70)**	**.033**
	Neonatal risk factors	**2.45**	**(1.07, 5.60)**	**.033**	**2.58**	**(1.28, 5.16)**	**.008**	1.04	(0.90, 1.18)	.601	**2.67**	**(1.31, 5.44)**	**.007**
Economical violence	Pregnancy risk factors	0.77	(0.25, 2.35)	.649	1.54	(0.78, 3.01)	.207	1.11	(0.86, 1.40)	.412	1.70	(0.80, 3.62)	.167
	Delivery risk factors	1.78	(0.49, 6.28)	.375	1.87	(0.99, 3.51)	.051	0.94	(0.77, 1.13)	.510	1.75	(0.93, 3.29)	.080
	Neonatal risk factors	2.05	(0.99, 4.22)	.052	**1.99**	**(1.06, 3.73)**	**.030**	0.95	(0.83, 1.07)	.425	**1.89**	**(1.01, 3.52)**	**.043**
Sexual violence	Pregnancy risk factors	0.63	(0.15, 2.50)	.510	1.15	(0.53, 2.46)	.715	1.16	(0.78, 1.70)	.458	1.33	(0.56, 3.15)	.513
	Delivery risk factors	1.89	(−11.76, 0.30)	.492	1.41	(0.56, 3.53)	.462	0.87	(0.57, 1.32)	.515	1.23	(0.52, 2.89)	.639
	Neonatal risk factors	1.69	(0.63, 4.38)	.300	1.50	(0.64, 3.51)	.346	0.83	(0.60, 1.13)	.237	1.25	(0.54, 2.83)	.598

Boldface indicates a statistically significant result (*P* < .05).

CDE = controlled direct effect, CI = confidence interval, MTE = marginal total effect, OR = odds ratio, PDE = pure direct effect 3, TIE = total indirect effect.

The odds ratio of pure direct effect and marginal total effect of severe maternal morbidity increased by social violence during pregnancy risk factors by 2.54 (*P* = .024) and 2.65 (*P* = .013), respectively. The pure direct effect and marginal total effect of social violence through delivery risk factors increased the odds of severe maternal morbidity by 2.67 (*P* = .023) and 2.24 (*P* = .033), respectively. The odds ratio of direct effects (controlled and pure), and the marginal total effect of social violence through neonatal risk factors increased the risk of severe maternal morbidity by 2.45 (*P* = .033), 2.58 (*P* = .008), and 2.67 (*P* = .007), respectively. These significant effects demonstrated a direct association between social violence and maternal morbidity. The odds ratio of pure direct effect and marginal total effect are 1.99 (*P* = .030) and 1.89 (*P* = .043), respectively, for economic violence as exposure, neonatal risk factors as a mediator, and severe maternal morbidity as the outcome.

The excess relative risk of the reference interaction between physical violence and pregnancy risk factors is 3.94 (*P* = .022), indicating that this interaction increases the risk of severe maternal morbidity by 394% (Table 3).

**Table 3:** The results of mediation analysis for violence, its subgroup, and severe maternal morbidity with Med4way package.

		TE			INT REF			INT MED			PIE		*P*-value
		ERR	95% CI	*P*-value	ERR	95% CI	*P*-value	ERR	95% CI	*P*-value	ERR	95% CI	
Total violence	Pregnancy risk factors	0.40	(−0.71, 1.51)	.478	3.20	(−0.98, 7.39)	.134	−0.57	(−1.53, 0.40)	.250	0.85	(−0.29, 1.99)	.144
	Delivery risk factors	0.10	(−0.57, 0.77)	.774	0.07	(−1.97, 2.12)	.945	0.01	(−0.21, 0.23)	.945	−0.11	(−0.32, 0.10)	.314
	Neonatal risk factors	0.32	(−0.46, 1.10)	.426	−0.24	(−0.67, 0.18)	.268	−0.05	(−0.21, 0.11)	.525	−0.01	(−0.07, 0.05)	.732
Mental violence	Pregnancy risk factors	-0.20	(−0.80, 0.40)	.517	1.38	(−1.49, 4.25)	.348	−0.15	(−0.56, 0.25)	.455	0.33	(−0.25, 0.91)	.270
	Delivery risk factors	−0.32	(−0.74, 0.10)	.143	0.64	(−1.20, 2.49)	.496	−0.04	(−0.20, 0.12)	.612	0.08	(−0.15, 0.31)	.494
	Neonatal risk factors	−0.33	(−0.74, 0.07)	.114	−0.10	(−0.39, 0.19)	.503	−0.03	(−0.12, 0.06)	.587	−0.02	(−0.08, 0.05)	.623
Physical violence	Pregnancy risk factors	1.30	(−0.41, 3.02)	.138	**3.94**	**(0.57, 7.31)**	**.022**	−0.29	(−2.83, 0.25)	.101	1.32	(−0.04, 2.96)	.058
	Delivery risk factors	0.89	(−0.40, 2.19)	.178	0.54	(−2.87, 3.97)	.755	0.04	(−0.22, 0.29)	.776	−0.09	(−0.34, 0.16)	.505
	Neonatal risk factors	1.15	(−0.38, 2.68)	.142	−0.32	(−1.14, 0.50)	.448	−0.10	(−0.34, 0.24)	.574	−0.04	(−0.15, 0.07)	.485
Social violence	Pregnancy risk factors	1.66	(−0.37, 3.96)	.110	0.43	(−0.19, 7.06)	.064	−1.13	(−2.72, 0.45)	.162	1.24	(−0.12, 2.60)	.074
	Delivery risk factors	1.24	(−0.42, 2.90)	.074	−2.23	(−10.24, 5.77)	.585	−0.30	(−10.24, 5.77)	.585	−0.13	(−0.35, 0.08)	.230
	Neonatal risk factors	1.63	(−0.22, 3.48)	.085	−0.18	(−1.25, 1.01)	.839	0.03	(−0.26, 0.32)	.844	0.05	(−0.05, 0.19)	.467
Economical violence	Pregnancy risk factors	0.71	(−0.58, 1.99)	.281	1.55	(−1.89, 5.00)	.376	−0.13	(−0.56, 0.29)	.536	0.30	(−0.40, 1.00)	.405
	Delivery risk factors	0.75	(−0.35, 1.86)	.180	0.70	(−4.06, 2.66)	.683	−0.05	(−0.30, 0.20)	.716	−0.07	(−0.25, 0.11)	.465
	Neonatal risk factors	0.83	(−0.29, 1.96)	.148	−0.22	(−0.77, 0.33)	.441	−0.06	(−0.24, 0.13)	.552	−0.04	(−0.14, 0.06)	.437
Sexual violence	Pregnancy risk factors	0.33	(−0.18, 1.48)	.566	1.50	(−1.48, 4.48)	.325	−0.24	(−0.90, 0.41)	.469	0.42	(−0.39, 1.23)	.308
	Delivery risk factors	0.23	(−0.82, 1.28)	.671	−1.25	(−6.40, 3.90)	.635	−0.11	(−0.61, 0.39)	.675	−0.07	(−0.25, 0.10)	.425
	Neonatal risk factors	0.17	(−0.77, 1.12)	.717	−0.20	(−0.77, 0.38)	.502	−0.13	(−0.54, 0.28)	.529	−0.10	(−0.25, 0.06)	.233

Boldface indicates a statistically significant result (*P* < .05).

CI = confidence interval, ERR = excess relative risk, INT MED = mediated interaction, INT REF = reference interaction, PIE = pure indirect effect, TE = total effect.

## 4. Discussion and conclusion

### 4.1. Interpretation of results

The interpretation of results as follows:

#### 4.1.1. Controlled direct effect

The controlled direct effect: the physical violence on severe maternal morbidity, mediated by neonatal risk factors, indicates that when neonatal risk factors are held constant (adjusted or controlled), the odds of severe maternal morbidity increase by 2.425 for physical violence and 2.456 for social violence.

#### 4.1.2. Pure direct effect

The pure direct effect of physical violence on severe maternal morbidity, mediated by pregnancy, and neonatal risk factors, shows that in women who experienced physical violence but did not have any pregnancy or neonatal risk factors (equal to 0), the odds ratio for severe maternal morbidity was 2.263 and 2.289, respectively.

The pure direct effect of social violence on severe maternal morbidity, mediated by pregnancy, delivery, and neonatal risk factors, shows that in women who experienced social violence but did not have any pregnancy, delivery, or neonatal risk factors (equal to 0), the odds ratio for severe maternal morbidity was 2.542, 2.673, and 2.577, respectively.

The pure direct effect of economic violence on severe maternal morbidity, mediated by pregnancy, delivery, and neonatal risk factors, showed that in women who experienced economic violence but did not have any neonatal risk factors (equal to 0), the odds ratio for severe maternal morbidity was 1.993.

#### 4.1.3. Marginal total effect

The marginal total effect of physical violence on severe maternal morbidity, mediated by pregnancy, and neonatal risk factors, indicates that the direct and indirect effects of physical violence through pregnancy and neonatal risk factors increase the odds of severe maternal morbidity by 2.296 and 2.151, respectively.

The marginal total effect of social violence on severe maternal morbidity, mediated by pregnancy, delivery, and neonatal risk factors, indicates that the direct and indirect effects of social violence through pregnancy, delivery, and neonatal risk factors increase the odds of severe maternal morbidity by 2.651, 2.239, and 2.673, respectively.

The marginal total effect of economic violence on severe maternal morbidity, mediated by neonatal risk factors, indicates that the direct and indirect effects of economic violence through neonatal risk factors increase the odds of severe maternal morbidity by 1.894.

#### 4.1.4. Reference interaction

The reference interaction between physical and social violence and pregnancy risk factors increased the risk of severe maternal morbidity by 394.6% and 360%, respectively (even after interaction, we prevent physical and social violence as exposure). It is important to note that the difference between the pure and controlled direct effects is as follows. In the first case, we assume that there are no mediators that can be caused by exposure. In the second case, the mediator is assumed to be fixed at a certain value in the population, which can be either the reference value or another value.^[15,18,19]^

This study utilized mediation analysis with the counterfactual approach to evaluate the association between domestic violence and its subgroup on severe maternal morbidity mediated by pregnancy, delivery, and neonatal risk factors.

Most studies have reported the total effect. For example, in a systematic review study by Taft, social determinants and intimate partner violence (physical violence, sexual violence, stalking, and psychological aggression) hurt maternal health during pregnancy and are associated with maternal mortality and severe maternal morbidity.^[5]^

### 4.2. Discussion

A case–control study reported a significant total effect between intimate partner violence on severe maternal morbidity.^[6]^ This study emphasizes that violence has a significant impact on pregnancy. In the current study, we found that social and physical violence had a direct effect on severe maternal morbidity (not the total and indirect effects). Total, sexual, economic, and psychological violence did not show a significant odds ratio in our study due to the small sample size. However, these studies did not consider mediation analyses or subgroups of violence. The interaction between social and physical violence (exposure) and pregnancy risk factors (mediators) was not discovered in those studies. although postpartum depression was significantly associated with increased odds of MNM/SMM (odds ratio: 1.83; 95% confidence interval 1.37–2.44).^[20]^

Vilma E conducted a survey of 6143 women who had live births between 1993 and 1995 in South Carolina.^[21]^ The prevalence rate of physical violence was 11.1%. Women who experienced physical violence were more likely to have a cesarean section and were hospitalized before delivery for maternal complications. In our study, physical violence had a direct effect on severe maternal morbidity and interacted with pregnancy risk factors.

Faramrzi reported a high prevalence of physical, emotional, and sexual violence (9.1%, 30.8%, and 19.2% in 3275 participants).^[22]^ They reported an association between adverse maternal and neonatal health (such as pregnancy morbidities) and violence. In this study, we also report a direct association between physical violence and severe maternal morbidity and its interaction with pregnancy risk factors.

Vilma E also showed that mothers who were exposed to physical violence in the year before childbirth had higher odds of hospitalization, maternal morbidities, and cesarean section.^[21]^ Another study reported an association between intimate partner violence and maternal morbidities. This study noted that mothers who were exposed to violence before and during the pregnancy period experienced weaker maternal and neonatal health.^[23]^

The controlled direct effect, pure direct effect, total effect, and interaction were significant in this study, indicating a generally direct association between exposure and outcome. Physical, social, and economic violence directly increases the risk of severe maternal morbidity. Additionally, the interaction between physical and social violence and pregnancy risk factors increases the risk of severe maternal morbidity.

Therefore, by using mediation analysis, researchers can identify the role of exposure and other variables in the occurrence of the outcome more effectively and, ultimately, prevent severe maternal morbidity by changing health policies.

The strengths of this study include utilizing a case–control study design, using the WHO approach to select cases and controls, employing a valid and reliable questionnaire to measure violence, and using a mediation analysis.

This study has several limitations, including a small sample size and possible information bias. This bias may arise from participants potentially providing dishonest responses when they complete the violence questionnaire.

It is recommended that researchers use larger sample sizes. Descriptive studies are suggested to collect details on violent information. Additionally, collecting a scale of variables suitable for using mediation analysis packages is preferable.

### 4.3. Conclusion

In summary, while this study contributes to understanding the association between domestic violence and severe maternal morbidity, caution should be exercised when generalizing these results to other settings or populations due to the small sample size, potential biases, and differences in analytical approaches. Further research with larger and more diverse samples is needed to enhance the generalizability of these findings across different healthcare contexts and demographic groups.

## Author contributions

**Conceptualization:** Sedigheh Abdollahpour.

**Data curation:** Sedigheh Abdollahpour, Hamid Heidarian Miri.

**Formal analysis:** Mahdieh Sahebi, Hamid Heidarian Miri.

**Methodology:** Mahdieh Sahebi, Masoumeh Sadeghi, Hamid Heidarian Miri.

**Supervision:** Hamid Heidarian Miri.

**Validation:** Hamid Heidarian Miri.

**Writing – original draft:** Mahdieh Sahebi, Sedigheh Abdollahpour, Masoumeh Sadeghi, Hamid Heidarian Miri.

**Writing – review & editing:** Mahdieh Sahebi, Sedigheh Abdollahpour, Masoumeh Sadeghi, Hamid Heidarian Miri.

## Supplementary Material


