# Supplementary material for: Direct and indirect association of domestic violence against women and severe maternal morbidity: A case–control study
Source: Medicine (Baltimore). 2025 Jan 24;104(4):e41268. doi: 10.1097/MD.0000000000041268 (PMC11771667; doi:10.1097/MD.0000000000041268)
Supplement: Supplementary file 1 [file medi-104-e41268-s001.docx]

Sup1: Definition of Causal Mediation Effects

We can estimate these effects with Paramed and Med4way packages:

The effects which can calculate from med4way and paramed packages in Stata

| Med4way | paramed |
| --- | --- |
| Controlled direct effect | Controlled direct effect |
| Reference interaction | Pure direct effect |
| Mediated interaction | Total indirect effect |
| Pure indirect effect | Marginal total effect |
| Total effect |  |

Effects were reported as odds ratios in the Paramed package and excess relative risk in the Med4way package.

Controlled Direct Effect (CDE):

The effect of violence on severe maternal, if pregnancy, delivery, and neonatal risk factors are fixed at a particular value. In this study, we fixed mediators at the reference level.

Pure Direct Effect (PDE):

The effect of violence on severe maternal morbidity if violence does not lead to any of the mediators (pregnancy, delivery, and neonatal risk factors).

Total Direct Effect (TDE):

The effect of violence and its interaction with pregnancy, delivery, and neonatal risk factors on severe maternal morbidity. If there is no interaction, the pure direct effect and the total direct effect are equal.

Pure Indirect Effect (PIE):

The effect of pregnancy, delivery, and neonatal risk factors (mediators) on severe maternal morbidity. Violence is controlled after creating pregnancy, delivery, and neonatal risk factors(mediators). We only estimated the effects of mediators on severe maternal morbidity.

Total Indirect Effect (TIE):

The effect of pregnancy, delivery, and neonatal risk factors and their interaction with violence on severe maternal morbidity. Violence is controlled after creating pregnancy, delivery, and neonatal risk factors and interactions. If there is no interaction, the pure indirect effect and the total indirect effect are equal.

Marginal Total Effect (MTE):

Direct and indirect effects of violence on severe maternal morbidity. This effect is the total effect that considers the counterfactual approach and is the product of the total indirect effect and pure direct effect.

Reference Interaction:

Effect of interaction between violence and pregnancy, delivery, and neonatal risk factors on severe maternal morbidity.

Mediated Interaction:

The effect of interaction between violence and pregnancy, delivery, and neonatal risk factors on severe maternal morbidity and the effect of violence on severe maternal complications through pregnancy, delivery, and neonatal risk factors(interaction and mediation)(13, 16).

Supp2. Distribution of Cases and Controls Based on Pregnancy, Delivery, and Neonatal Risk Factors (Mediators)

| **variables** | | **Cases (123)** | | | **Controls (127)** | | |
| --- | --- | --- | --- | --- | --- | --- | --- |
|  |  | **Number** | **Percent(%)** | | **Number** | **Percent(%)** | |
| Pregnancy risk factors | yes | 66 | | 53.66 | 18 | | 14.29 |
|  | no | 57 | | 46.34 | 108 | | 85.71 |
| Delivery risk factors | yes | 49 | | 39.84 | 19 | | 14.96 |
|  | no | 74 | | 60.16 | 108 | | 14.96 |
| Neonatal risk factors | yes | 93 | | 75.61 | 80 | | 62.99 |
|  | no | 30 | | 24.39 | 47 | | 37.01 |

Supp3. Distribution of Case and controls Based on Violence and Its Subgroup

| **Violence subgroup** | | **Cases(123)** | | **Controls(127)** | |
| --- | --- | --- | --- | --- | --- |
|  |  | **frequency** | **Percent(%)** | **frequency** | **Percent(%)** |
| Total violence | yes | 63 | 51.22 | 60 | 48.78 |
|  | no | 60 | 48.78 | 66 | 51.97 |
| Mental violence | yes | 53 | 43.09 | 57 | 44.88 |
|  | no | 70 | 56.91 | 70 | 55.12 |
| Physical violence | yes | 37 | 30.08 | 24 | 18.90 |
|  | no | 86 | 69.92 | 103 | 81.10 |
| Social violence | yes | 84 | 68.29 | 21 | 16.54 |
|  | no | 39 | 31.71 | 106 | 83.46 |
| Economical violence | yes | 56 | 45.53 | 52 | 40.94 |
|  | no | 67 | 54.47 | 75 | 59.06 |
| Sexual violence | yes | 21 | 17.7 | 21 | 16.54 |
|  | no | 102 | 82.93 | 106 | 83.46 |
